# 3.0 T MRI IVIM-DWI for predicting the efficacy of neoadjuvant chemoradiation for locally advanced rectal cancer

**DOI:** 10.1007/s00261-020-02594-4

**Published:** 2020-05-27

**Authors:** Hongbo Hu, Huijie Jiang, Song Wang, Hao Jiang, Sheng Zhao, Wenbin Pan

**Affiliations:** 1 Department of Radiology, The Second Affiliated Hospital of Harbin Medical University, Harbin, 150086 China; 2 Department of Radiology, Longhua Hospital, Shanghai University of Traditional Chinese Medicine, No. 725, South Wanping Road, Shanghai, 200032 China

**Keywords:** Rectal cancer, Magnetic resonance imaging, Intravoxel incoherent motion diffusion-weighted imaging, Neoadjuvant chemoradiotherapy

## Abstract

**Purpose:**

The purpose of this study was to determine the diagnostic performance of intravoxel incoherent motion (IVIM) on assessing response to neoadjuvant chemoradiation (nCRT) in patients with Locally Advanced Rectal Cancer (LARC).

**Methods:**

50 patients with rectal cancer who underwent magnetic resonance (MR) imaging before and after nCRT, the values of pre-nCRT and post-nCRT IVIM-DWI parameters apparent diffusion coefficient (ADC), diffusion coefficient (*D*), false diffusion coefficient (D*), and perfusion fraction (*f*), together with the percentage changes (∆% parametric value) induced by nCRT were calculated. According to the patient's response to nCRT, the patients were divided into pathological complete response (pCR) and non-pCR groups, Good Response (GR) group and Poor Response (PR) group, and the above values were compared between different groups. Univariate and multiple logistic regression analysis were done to investigate the relation between different parameters and patient nCRT. Draw ROC curve according to sensitivity and specificity, and compare its diagnostic efficacy.

**Results:**

There were no significant differences in the baseline data of 50 patients. After nCRT, the ADC and D values for LARC increased significantly (all *p* < 0.05). The pCR group (*n* = 9) had higher preD*, pre*f*, postD*, ∆%ADC and ∆%*D* values than the non-pCR group (*n* = 41) (all *p* < 0.05). The GR group (*n* = 17) exhibited higher post D, ∆%ADC and ∆%*D* values than the PR group (*n* = 33) (all *p* < 0.05). From the results of Logistic regression analysis found that ∆%ADC and ∆%*D* were significantly correlated with patients' response to nCRT. Based on ROC analysis, ∆%*D* had a higher area under the curve value than ∆%ADC (*p* = 0.009) in discriminating the pCR from non-pCR groups.

**Conclusions:**

IVIM-DWI technology may be helpful in identifying the pCR and GR patients to nCRT for LARC.

## Introduction

Colorectal cancer (CRC) is one of the most common malignancies with high morbidity and mortality in the world. In recent years, the incidence of colorectal cancer in China has increased year by year, 60–70% of these cases are of locally advanced rectal cancer (LARC) [1, 2]. LARC includes those cases in which the tumor has spread beyond the wall of the rectum into the surrounding perirectal fat by at least 5 mm (T3c–d), when the tumor has invaded local adjacent structures (T4), or when there is involvement of locoregional lymph nodes (N1 or N2) and without distant metastasis (M0), and tumor within 12 cm from the anal verge [3, 4].

Neoadjuvant chemoradiotherapy (nCRT) followed by total mesorectal excision (TME) has become a standard treatment in patients with locally advanced rectal cancer (LARC), which could decrease the loco-regional recurrence rate and even increase overall survival. However, most patients with rectal cancer in China are LARC at the first diagnosis. The current clinical data show that after nCRT and other neoadjuvant therapy, about 20% of rectal cancer patients can even achieve pathological complete response [5, 6]. However, not all patients can benefit from nCRT, tumor downstaging in patients with LARC, tumor regression differs from patient to patient, ranging from pCR, intermediate regression, or even a complete lack of response. Early detection in poor responders to nCRT could provide an opportunity for these patients to proceed directly to surgery, thereby avoiding the morbidity associated with nCRT or to intensive treatment regimens such as second-line chemotherapy or a higher radiation dose to maximize the therapeutic response [7]. Inappropriate treatment will not only delay the operation time, but also increase the risk of distant metastasis.

Imaging studies are frequently used to evaluate patients for screening and staging of colorectal cancer. A recent meta-analysis demonstrated that MRI had inconsistent results in diagnostic performance for restaging rectal cancer after neoadjuvant treatment [8]. Better results were demonstrated when using diffusion-weighted imaging and/or observers with > 5 years’ experience reading rectal/pelvic MRI. DWI can be used to observe the random movement of water molecules in living tissues, and it is widely accepted in the clinic because of its non-invasive nature [9]. DWI and its quantitative parameter ADC, which reflects the spread of rectal cancer, were widely studied by researcher at home and abroad due to their value of predicting and evaluating the curative effect of nCRT for colorectal cancer, but the results are not accepted by everyone. However, in fact, the free motion of water molecules in the human body is affected by many factors and is a non-Gaussian motion, so the ADC value cannot reflect the diffusion of water molecules truly [10]. Some researchers have proposed a more scientific intravoxel incoherent motion model to supplement it [11–13]. The IVIM-DWI is based on the multi-b-valued DWI sequence, and the parameters reflecting microvascular perfusion and diffusion of water molecules in living tissues can be obtained using double exponential model, including true diffusion coefficient (*D*), false diffusion coefficient (D*), and perfusion fraction (*f*). The distinction between true and false perfusion in IVIM-DWI makes up for the deficiency of DWI, reflects the microcirculation perfusion in the capillary network. Therefore, the purpose of this study was to explore the feasibility of IVIM-DWI predicting the effect of nCRT in LARC patients, and analyze whether the distribution of quantitative parameters is different between pCR and non-pCR patients before and after neoadjuvant radiochemotherapy, so as to provide the possibility for follow-up study. Then guide the clinical further screening of colorectal cancer patients suitable for nCRT, to provide more reference for the setting of the individualized treatment plan in the context of multidisciplinary comprehensive treatment, so that patients can benefit more from the treatment.

## Materials and methods

### Subject

This study was reviewed by the ethics committee of our institution, and informed consent was obtained from all patients. This study retrospectively analyzed the data of 50 patients of LARC diagnosed and treated in the hospital from May 2018 to February 2019, including 32 males and 18 females, aged 36 to 63 years, the average age was (48.4 ± 15.0) years. The inclusion criteria were: (1) Patients with rectal cancer confirmed by colonoscopy biopsy. (2) Patients diagnosed as LARC by imaging examination before treatment. (3) Patients undergoing surgical treatment after nCRT as required in this study. The exclusion criteria were: (1) Patients who received other antineoplastic therapy before nCRT. (2) Incomplete clinical data (3) Patients who fail to complete nCRT treatment. (4) Patients who have not been able to complete the operation.

### Experimental equipment and conventional rectal MRI sequence

All patients underwent conventional MRI examinations and IVIM-DWI. The a GE Discovery MR750w 3.0 T MRI scanner was used to collect the image data by using the phased array body coil, and the conventional sequence and multi-b value DWI sequence were scanned, and the related parameter values were obtained by Function Tool post-processing software analysis. The imaging parameters are summed up in Table 1.

**Table 1:** Conventional rectal MRI sequence

Parameter	T1WI flair	T2WI propeller	T2WI fs propeller	T2WI propeller	T2WI propeller	IVIM sequence
Acquisition plane	Axial	Axial	Axial	Sagittal	Coronal	Axial
Repetition time (ms)	460	5230	5400	6150	4700	2000
Echo time (ms)	23	77	77	72	77	80
Slice thickness (mm)	5	5	5	5	5	5
Slice gap (mm)	1	1	1	1	1	1
Field of view (mm^2^)	250 × 250	250 × 250	250 × 250	270 × 270	250 × 250	420 × 420
Number of layers	24	24	24	24	18	24
Number of excitations	2	2	2	2	2	2
Scanning time	2 min 20 s	1 min 50 s	1 min 35 s	2 min 10 s	1 min 19 s	5 min 48 s

### Preparation and scanning methods before the examination

Before the examination, confirm that the patient is stable and free of contraindications (no metal implants, no claustrophobia, etc.). Explain to the patients before the examination and patients' informed consent. At least half an hour before the examination, the bowel was cleaned without air or any contrast agent. All patients underwent rectal MRI scan and multi-*b* value DWI examination, no enhanced examination.

### Multi-*b*-valued DWI sequence

The b values of axial DWI sequence were set at 10, and 50, 100, 150, 200, 400, 600, 1000, 1500, 2000, 0 s/mm^2^.

### DWI image post-processing

**Fig. 1:**
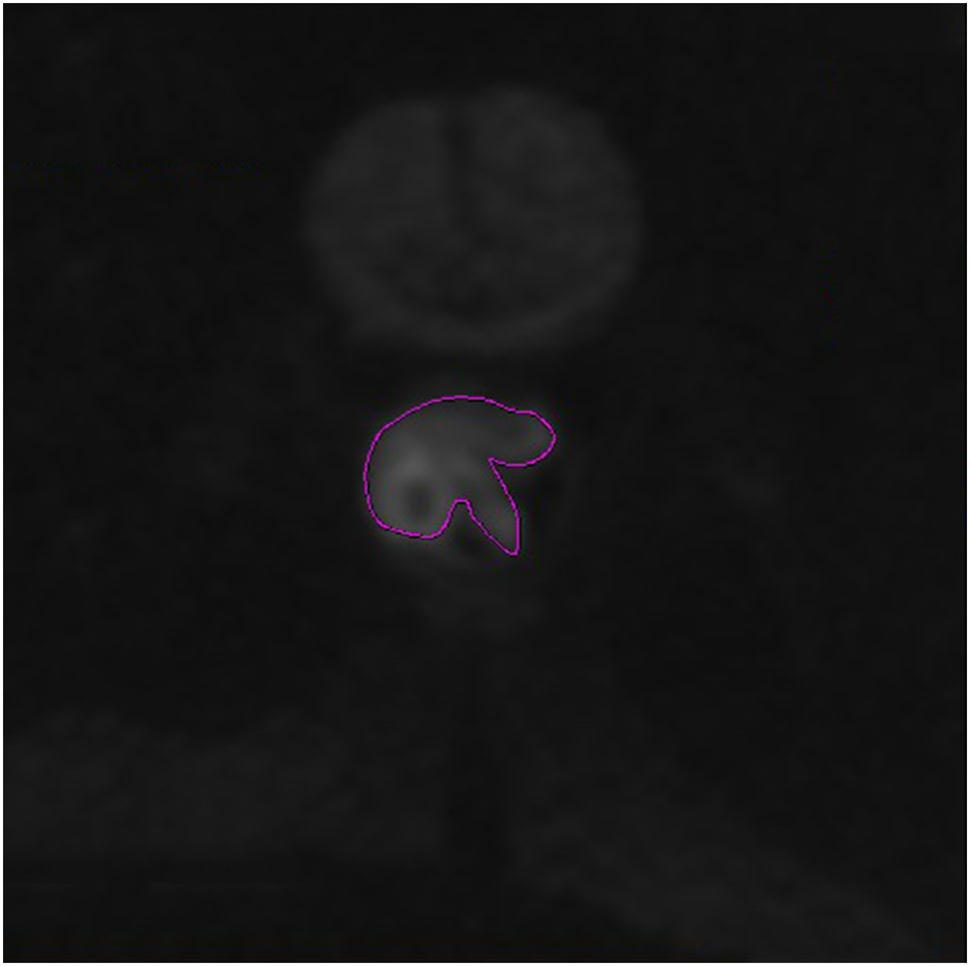
Image post processing. Dotted lines show the tumor border

**Fig. 2:**
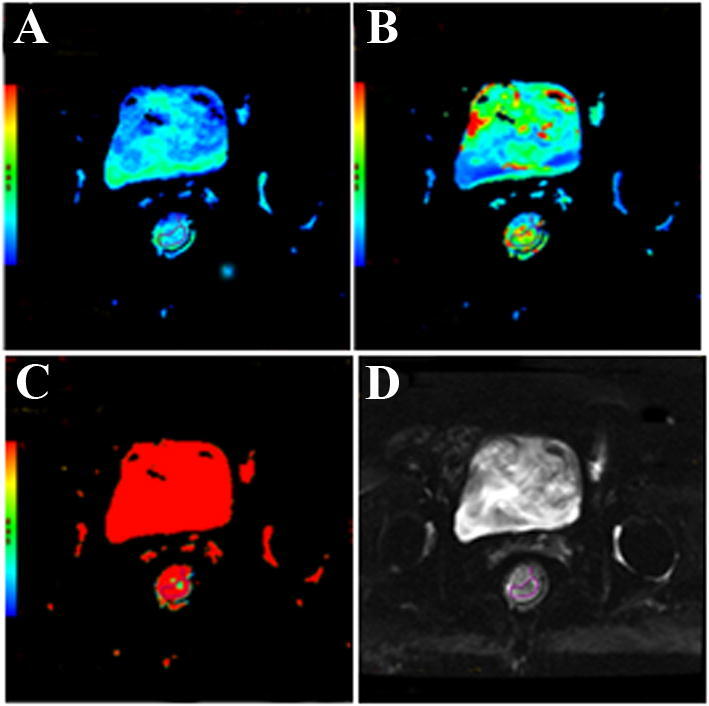
GE Function Tool post-processing software, image post-processing: delineate the region of interest, measure the quantitative parameters. **a** *D*: pure diffusion coefficient; **b** D*: pseudo-diffusion coefficient; **c** *f*: perfusion fraction; **d** ADC map: apparent diffusion coefficient

Two radiologists with 9 and 12 years of experience in abdominal MRI process the images before and after treatment. Image processing using GE Function Tool post-processing software after scanning (Fig. 1), the ADC images based on the single exponential model and the pseudo-color images based on the IVIM-DWI quantitative parameters *D*, D* and *f* of the double exponential model were selected. Diffusion correlation coefficient (*D*) and perfusion correlation coefficient (D*, *f*) of water molecules, so as to distinguish the diffusion movement of water molecules in vivo from microcirculation perfusion. The high signal lesion area is selected on the axial DWI image of *b* = 1000 s/mm^2^, and the corresponding level of T2WI image is used as the anatomic structure reference, which requires that the blood vessels, tumor necrosis and bleeding components should not be included, to avoid the influence of heterogeneous components on the measurement results. On the pseudo-color images of each parameter, the solid part of the tumor is selected to outline the region of interest at the maximum level of the tumor and its upper and lower levels (Fig. 2), and then the measurement results of the three-layer region of interest are averaged. The relatively reliable values of ADC (10^−3^mm^2^/s), *D* (10^−3^mm^2^/s), D* (10^−3^mm^2^/s) and *f* were obtained. IVIM uses double exponential model and multiple b values to fit and calculate on the basis of DWI, and obtains the diffusion correlation coefficient (*D*) and perfusion correlation coefficient (D*, *f*) of water molecules, so as to distinguish the diffusion movement of water molecules in vivo from microcirculation perfusion. The measured values of the two doctors and the mean value of the two persons were recorded, respectively. In the statistical analysis, the mean value of the two persons with the same observation was taken as the mean value.

### Evaluation criteria

The staging of rectal cancer was determined by referring to the 8th edition of TNM staging criteria published by the International Union against Cancer [14].

Pathological results: the patients without any adenocarcinoma cells in the surgical specimens are pCR, and the rest were non-pCR. The patients were divided into pCR group and non-pCR group according to the postoperative pathological results.

### Pathological response evaluation

TME was performed after post-nCRT MRI examinations. After TME, the fresh specimens were fixed in formalin for 48 h. Tissue sections stained with haematoxylin–eosin were evaluated by two pathologists. Postoperative tumor staging was performed according to the American Joint Committee on Cancer (AJCC) TNM system [15]. The pathologic response induced by nCRT was categorized according to the Dworak tumor regression grade (TRG) system as follows [16, 17]: TRG 4, absence of residual cancer, only a fibrotic mass; TRG 3, presence of rare residual cancer cells scattered through the fibrosis; TRG 2, increased number of residual cancer cells, but still predominating fibrosis; TRG 1, residual cancer outgrowing fibrosis; TRG 0, absence of regression changes. In this study, the patients with TRG 4 were categorized as the pathological complete responder (pCR) group, whereas the non-pathological responder (non-pCR) group consisted of those with other TRG scores. We also classified the patients into the GR (TRG 3 or 4) and PR (TRG 0, 1 or 2) groups.

### Univariate and multivariate analysis

In a univariate analysis, we used the univariate logistic regression analyses to compare the parameters (preADC, preD, preD*, pre*f*, postADC, postD, postD*, post*f*, ∆%ADC, ∆%D, ∆%D*) between the patients with pCR group and non-pCR group. If a statistical significance was obtained for more than two parameters among all parameters, these parameters were further analyzed by multivariate logistic regression models to determine whether they had independent predictive value with odds ratios and corresponding 95% confidence intervals.

### ROC analysis

We used the receiver operating curve (ROC) to evaluate the predictive power of each parameter, and the Youden index (sensitivity, 1-specificity) to determine the sensitivity, specificity and positive predictive value, negative predictive value and diagnosis of each parameter accuracy. *p* < 0.05 indicates that the difference is statistically significant.

### Statistical analysis

In this study, EXCEL 2013 was used to establish the database, and SPSS 22.0 statistical software was used to compare the data of the two groups of patients. The measurement data were expressed as $\overline{x} \pm s$. The comparison between the two groups was analyzed by *t-*test, the counting data was expressed by percentage (%). *p* < 0.05 indicated that the difference was statistically significant.

## Results

### Baseline data of patients included in the study

3.1.1. Four patients were excluded from this study due to lack of clinical information (*n* = 4). The cohort of this study eventually included 50 LARC patients. Table 2 lists the clinical characteristics of this cohort.

**Table 2:** Clinical characteristics of patients in the study

	Statistics
Age (years)	48.4 ± 15.0
Sex	
Male	32 (64.0%)
Female	18 (36.0%)
BMI	23.8 ± 3.9
Distance of the primary tumor from the anus	
0–5.0 cm	13 (26.0%)
5.1–10.0 cm	30 (60.0%)
10.1–15.0 cm	7 (14.0%)
Hypertension	
No	41 (82.0%)
Yes	9 (18.0%)
Diabetes mellitus	
No	39 (78.0%)
Yes	11 (22.0%)
Smoke	
Never smoking	36 (72.0%)
Smoking or smoked previously	14 (28.0%)
Drinking	
Never drinking	40 (80.0%)
Drinking or drank previously	10 (20.0%)
Post-nCRT pathologic T (ypT) classification	
ypT0	7
ypT1	14
ypT2	12
ypT3	9
ypT4	8
Pathological response to nCRT	
TRG 4	9
TRG 3	8
TRG 2	12
TRG 1	13
TRG 0	8

#### Comparison of IVIM-DWI parameters before and after nCRT in different groups

**Fig. 3:**
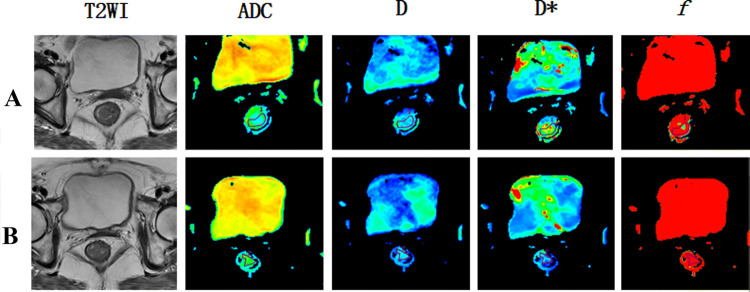
A patient with LARC from the pCR group. Images in sequence are pre- and post-therapy MR imaging, and IVIM-DWI parametric maps. *pCR* pathological complete response, *non-pCR* non-pathological complete response; *GR*, good response, *PR* poor response, *T2WI* T2-weighted imaging, *ADC* apparent diffusion coefficient, *D* pure diffusion coefficient, D* pseudo-diffusion coefficient; *f*: perfusion fraction

**Fig. 4:**
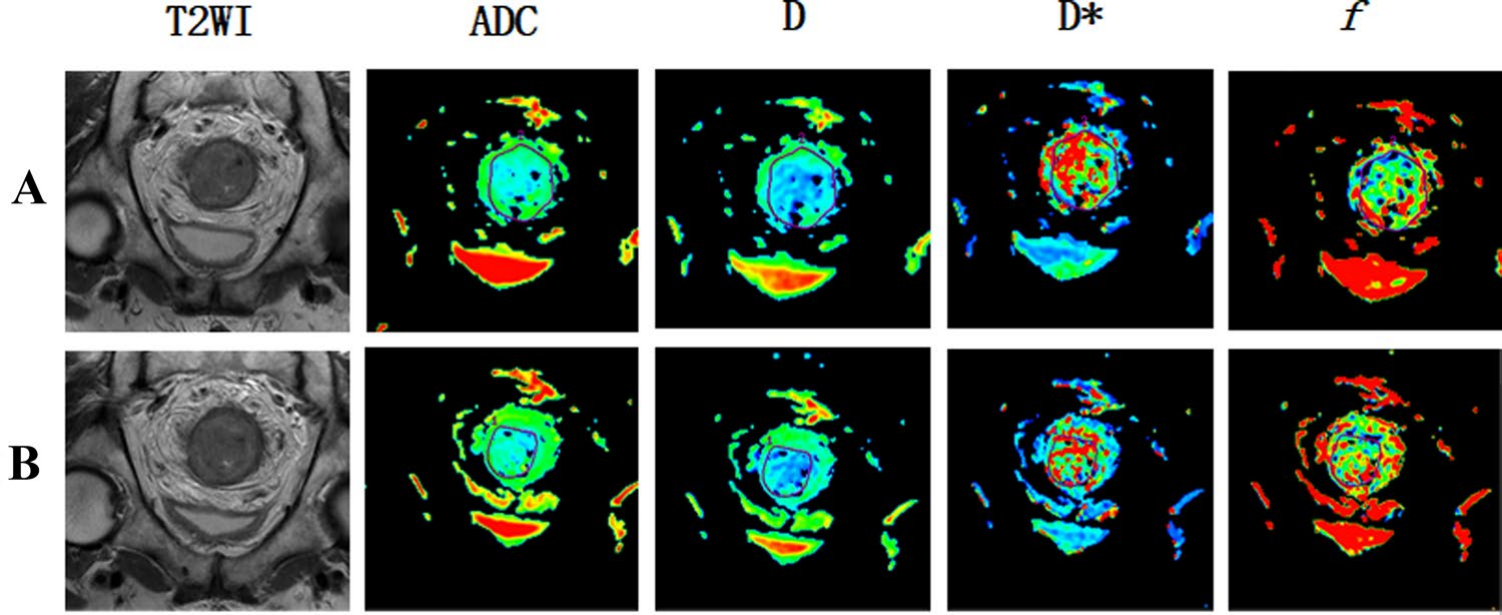
A patient with LARC from the non-pCR group. Images in sequence are pre- and post-therapy MR imaging, and IVIM-DWI parametric maps. *pCR* pathological complete response, *non-pCR* non-pathological complete response; *GR*, good response, *PR* poor response, *T2WI* T2-weighted imaging, *ADC* apparent diffusion coefficient, *D* pure diffusion coefficient, D* pseudo-diffusion coefficient; *f*: perfusion fraction

**Fig. 5:**
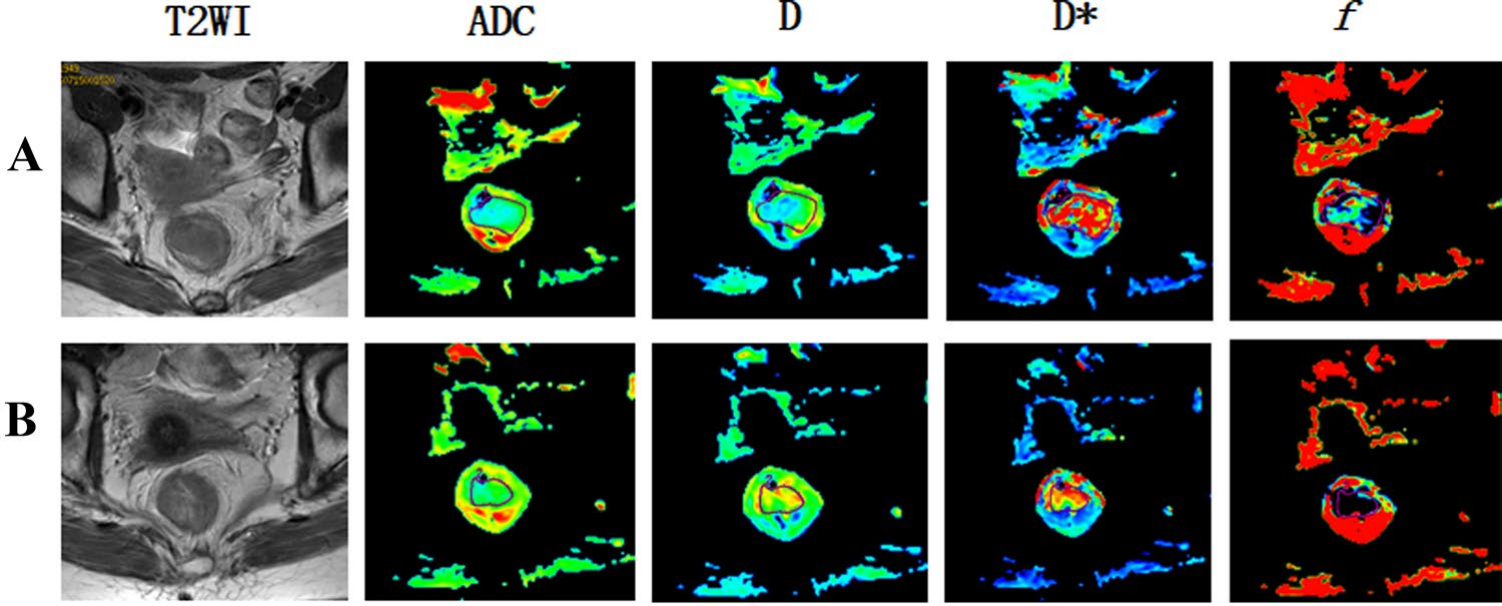
A patient with LARC from the GR group. Images in sequence are pre- and post-therapy MR imaging, and IVIM-DWI parametric maps. *pCR* pathological complete response, *non-pCR* non-pathological complete response; *GR*, good response, *PR* poor response, *T2WI* T2-weighted imaging, *ADC* apparent diffusion coefficient, *D* pure diffusion coefficient, D* pseudo-diffusion coefficient; *f*: perfusion fraction

**Fig. 6:**
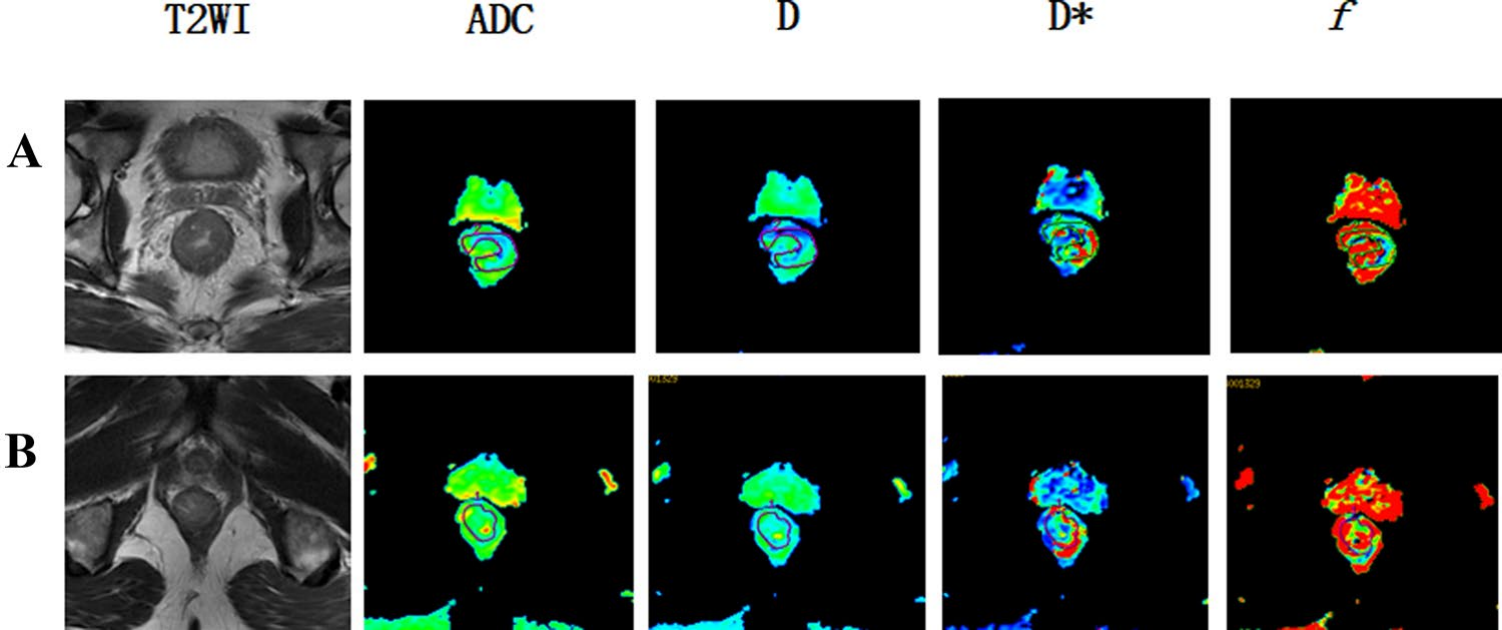
A patient with LARC from the PR group. Images in sequence are pre- and post-therapy MR imaging, and IVIM-DWI parametric maps. *pCR* pathological complete response, *non-pCR* non-pathological complete response; *GR*, good response, *PR* poor response, *T2WI* T2-weighted imaging, *ADC* apparent diffusion coefficient, *D* pure diffusion coefficient, D* pseudo-diffusion coefficient; *f* perfusion fraction

**Fig. 7:**
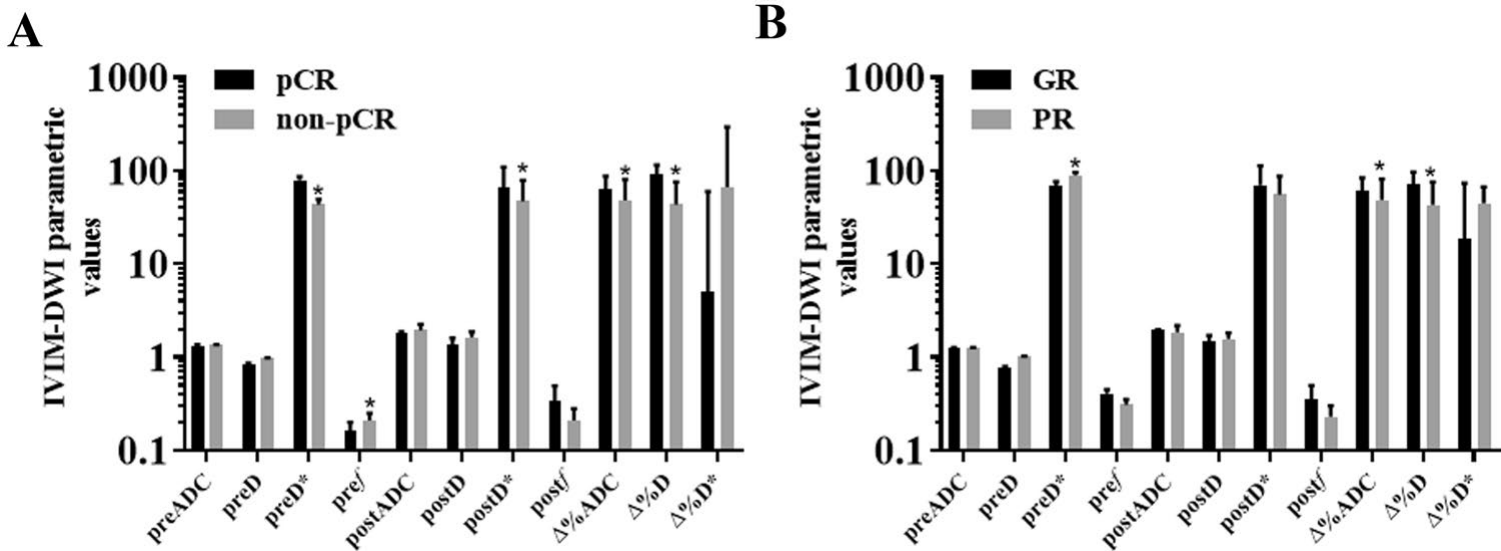
Differences in the IVIM-DWI parametric values between the pCR and non-pCR groups and between the GR and PR groups. **a** Difference in the IVIM-DWI parametric values for pCR and non-pCR; **b** Difference in the IVIM-DWI parametric values for GR and PR. 1. PreADC; 2. PreD; 3. PreD*; 4. Pre*f*; 5. PostADC; 6. PostD; 7. PostD*; 8. Post*f*; 9. ∆%ADC(%); 10. ∆%D(%); 11. ∆%D*(%). *GR* good response, *PR* poor response, *ADC* apparent diffusion coefficient, *D* pure diffusion coefficient; D* pseudo-diffusion coefficient, *f* perfusion fraction

Before nCRT, the patients were examined by MRI, and their IVIM-DWI related values (*D*, D*, ADC, *f*) were counted. Group patients according to pathological results after neoadjuvant chemotherapy: pCR group and non-pCR group, GR group and PR group. MRI was performed on them. The representative figures of each group are as shown in Fig. 3, 4, 5 and 6. The IVIM related values [*D* = (1.18 ± 0.18) × 10^–3^ mm^2^/s, D* = (0.89 ± 0.15) × 10^–3^ mm^2^/s, ADC = (43.45 ± 28.63) × 10^–3^ mm^2^/s and *f* = 0.23 ± 0.02] were counted, and the differences between before and after treatment [*D* =  (1.75 ± 0.28) × 10^–3^ mm^2^/s, D* = (1.29 ± 0.47) × 10^–3^ mm^2^/s, ADC = (48.57 ± 33.20) × 10^–3^ mm^2^/s and *f* = 0.22 ± 0.11] were calculated. Between pre-nCRT and post-nCRT, there were significant differences in the ADC and D values (all *p* < 0.001), whereas no significant differences were found in the D* and *f* values (*p* = 0.514 and 0.061, respectively). It was found that in the pCR and non-pCR groups, as well as GR and PR groups, ∆%D and ∆%ADC value were significant difference before and after treatment (*p* < 0.05). The results are as shown in Fig. 7.

#### Univariate and multivariate logistic regression analysis

It showed that the preD*, pre*f*, postD*, Δ%D and Δ%ADC values between the pCR group and the non-pCR group were statistically significant. Multiple logistic regression analysis showed that Δ%*D* value and Δ%ADC value were independent predictors of pCR (*p* = 0.034 and *p* = 0.009), where Δ%*D* value odd ratio was determined 2.42, the interval of 95% confidence is (0.02, 10.68). Δ% ADC value odd ratio was 5.89, the interval of 95% confidence is (2.56, 11.28). In contrast, the preD*, pre*f*, and postD* values indicate that it is not an important parameter to predict whether the pathology is completely resolved (all *p* > 0.05) (Table 3).

**Table 3:** Summarizes the results of univariate and multivariate logistic regression analysis

Parameter	Univariate analysis	
	Odds ratio	*P*
preADC	0.17 (0.012, 18.81)	0.069
preD	2.46 (0.02, 10.56)	0.11
preD*	6.56 (0.63, 18.13)	0.008*
pre*f*	4.47 (0.62, 15.1)	0.012*
postADC	0.21 (0.08, 0.69)	0.510
postD	3.13 (1.24, 17.52)	0.066
postD*	6.12 (3.58, 21.22)	0.002*
post*f*	3.66 (1.45, 16.54)	0.078
Δ%ADC	5.42 (0.52, 12.1)	0.023*
Δ%*D*	4.56 (4.2, 17.25)	0.028*
Δ%D*	0.52 (0.12, 0.51)	0.740

Parameter	Multivariate analysis	
	Odds ratio	*P*
preD*	0.74 (0.74, 1.05)	0.452
pref	0.05 (0.001, 24.5)	0.112
postD*	0.21 (0.08, 0.69)	0.343
Δ%ADC	5.89 (2.56, 11.28)	0.034*
Δ%*D*	2.42 (0.02, 10.68)	0.009*

Correlation between pCR and the IVIM-DWI parametric values

The odds ratio data in parentheses are 95% confidence intervals. P value with * mean statistical significance

*pCR* pathological complete response, *IVIM-DWI* Intravoxel incoherent motion diffusion-weighted imaging, *ADC* apparent diffusion coefficient, *D* pure diffusion coefficient, D* pseudo-diffusion coefficient, *f* perfusion fraction

#### ROC curve of IVIM-DWI parameters

**Fig. 8:**
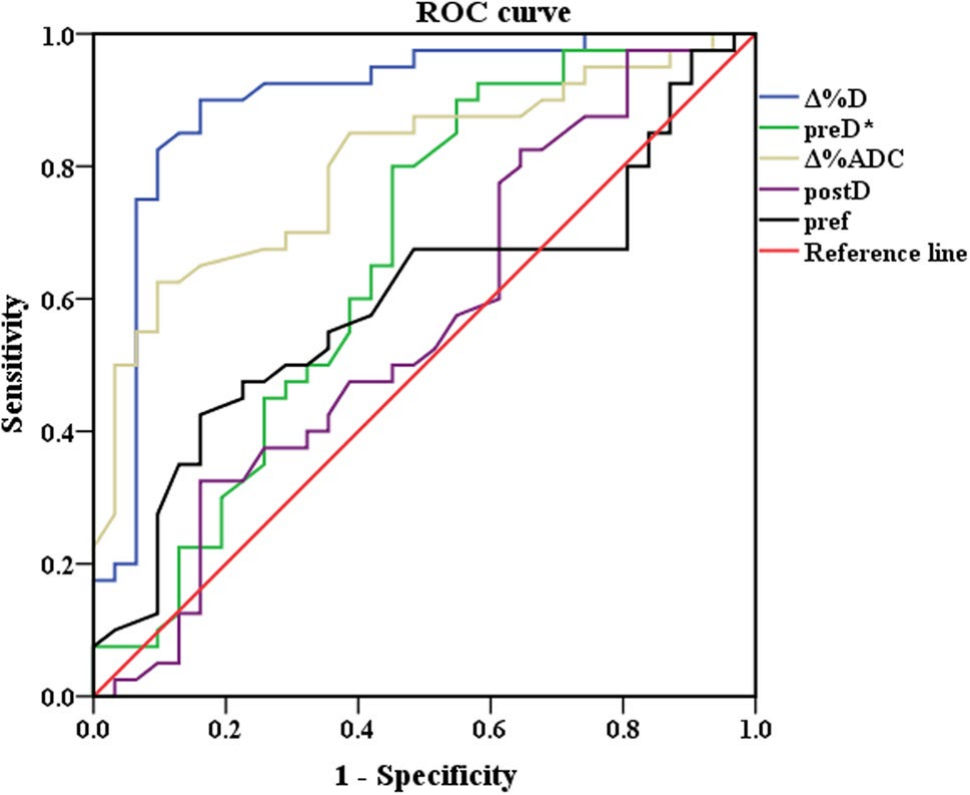
Receiver operating characteristic curve (ROC) for pCR group and non-pCR group in predicting response to neoadjuvant chemoradiation (nCRT). The area under the curve (AUC) was calculated for ROC curves, and sensitivity and specificity were calculated. The AUC is a measure of accuracy. The closer the curve follows the upper-left border of the ROC space, the more accurate the test. The closer the curve comes to the 45° diagonal of the ROC space, the less accurate the test

**Fig. 9:**
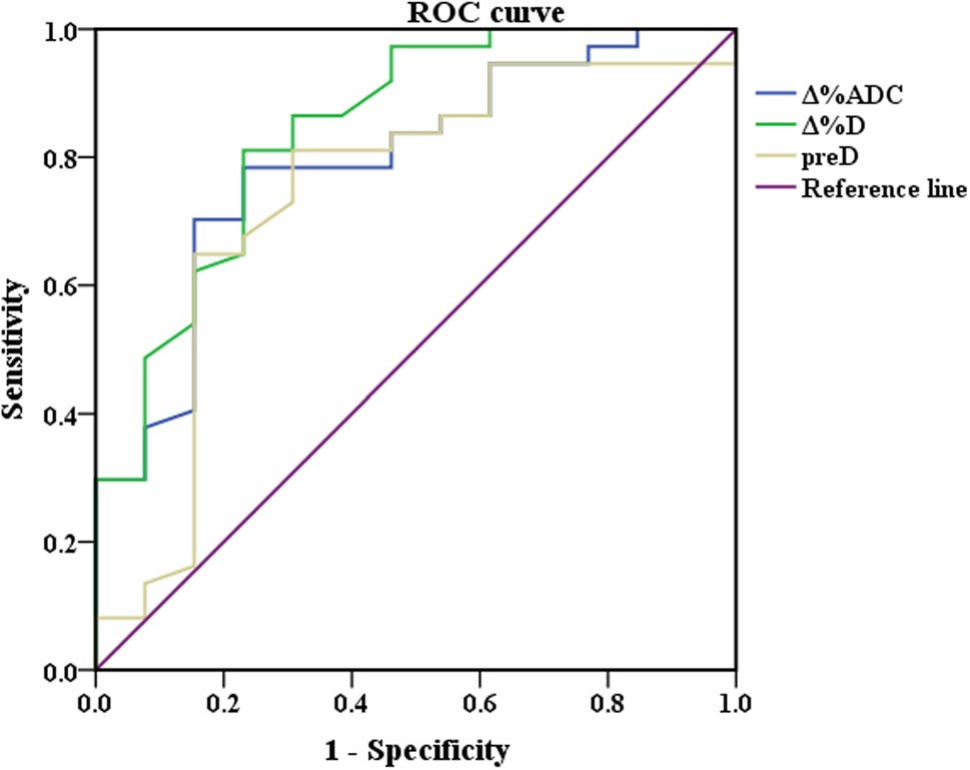
Receiver operating characteristic curve (ROC) for PR group and GR group in predicting response to neoadjuvant chemoradiation (nCRT). The area under the curve (AUC) was calculated for ROC curves, and sensitivity and specificity were calculated. The AUC is a measure of accuracy. The closer the curve follows the upper-left border of the ROC space, the more accurate the test. The closer the curve comes to the 45° diagonal of the ROC space, the less accurate the test

Based on ROC curve analysis, the diagnostic performance of the IVIM-DWI parameters in identifying pathological responses are shown in Figs. 8 and 9. To discriminate pCR from non-pCR, Δ%D had the highest area under the curve (AUC) (0. 898), sensitivity and positive predictive value among the five IVIM-DWI parameters (preD*, pre*f*, postD*, Δ%ADC and Δ%*D*), which could benefit the identification of pCR to nCRT. Among the three IVIM-DWI parameters (postD, Δ%ADC and Δ%*D*), which were helpful in distinguishing the GR from PR. The postD had the highest specificity and positive predictive value with an AUC of 0.793, whereas Δ%*D* had the highest sensitivity and negative predictive value with an AUC of 0.843.

## Discussion

MRI is currently an important method for preoperative staging of colorectal cancer [18–20]. It has high accuracy in the location and diagnosis of tumors, judgment of tumor infiltration depth, and determination of resection range. However, the biggest problem based on morphological evaluation is that it can only reflect the intuitive changes of cancer instead of reflect the information of the cell and molecular level accurately. IVIM uses double exponential model and multiple b values to fit and calculate on the basis of DWI, and obtains the diffusion correlation coefficient (*D*) and perfusion correlation coefficient (D*, *f*) of water molecules, so as to distinguish the diffusion movement of water molecules in vivo from microcirculation perfusion [21–23].

In this study, we focused on the diagnostic efficacy of IVIM-DWI in the evaluation of nCRT in the treatment of LARC, and we evaluated the therapeutic effect of patients through pathological results. In our 50 patient cohort, we found that between pre-nCRT and post-nCRT, there were significant differences in the ADC and D values. The diffusion coefficient (ADC) helps to assess the degree of water molecule diffusion limitation and is related to tumor proliferation, tumor necrosis and other factors [24–26]. Before nCRT, tumor cells proliferated massively, and the cell density increased significantly, which resulted in limited diffusion of water molecules so the ADC value was low [27]. In the macroscopic morphological level, the tumor volume shrinks and the T stage decreases when the nCRT reaches pCR; in the microscopic molecular level, the tumor cell membrane integrity is destroyed, permeability and necrosis is increased, density and extracellular space is reduced [28].

Our study also found that between the pCR group and the non-pCR group, the difference between preD*, pre*f*, postD*, Δ%*D* and Δ%ADC parameters is statistically significant. IVIM-DWI parameters D* and *f* reflect microvascular perfusion, which can reflect the proportion of false diffusion caused by microcirculation perfusion. The abundance the blood vessels, the greater the value of perfusion-related parameters. The D* and *f* values of the pCR group before treatment of LARC were higher than those of the non-pCR group, indicating that the lesions in the pCR group had higher microvascular perfusion. Similar studies have found that IVIM-DWI can be used to reflect the histopathological tumor regression grade after nCRT in LARC patients [29], patients with high *f* value before treatment had good tumor regression performance (specificity 100%). The value of ADC before treatment could not reflect the curative effect of nCRT.

The Δ%*D* and Δ%ADC value in the pCR group were also higher than that of the non-pCR group, indicating that the cell density of the lesions in the pCR group was smaller and the extracellular space was larger. We also proved that Δ%*D* and Δ%ADC are related to whether the patient's pCR and GR after treatment. They have higher sensitivity and specificity than other values. This discovery will be very meaningful, and it provides a potential independent predictor for clinical IVIM to predict the effect of neoadjuvant chemotherapy on LARC [29–31]. Bates et al. showed that diffusivity derived from the baseline staging MRI, but not diffusion kurtosis or volumetric data, is associated with TRG and therefore shows promise as a potential imaging biomarker to predict the response to neoadjuvant chemotherapy in LARC [32], however in our study we proved that Δ%*D* and Δ%ADC related to the TRG, which is a supplement to David's research.

Some limitations of our study need to be carefully considered. (1) The study cohort was small and the results are from a single institution. (2) Respiratory movement and intestinal peristalsis may affect the test results. Although respiratory gating is used, it is inevitable that there will be a slight displacement of organs. (3) Because IVIM-DWI parameters need to be fitted and calculated by DWI data with a wide range of *b* values, the stability of parameter measurement depends to a large extent on image quality, including signal-to-noise ratio (SNR) and position matching, etc. In the future, we can try to summarize the appropriate scanning methods in order to reduce the impact of scanning factors.

## Conclusion

In conclusion, IVIM technology can predict the efficacy of locally advanced rectal cancer by Δ%*D* and Δ%ADC value. They have a strong correlation with the pathology of patients after nCRT therapy.

## Data Availability

The datasets used and/or analysed during the current study are available from the corresponding author on reasonable request. Not applicable.
